# 2,2-Dichloro-1-[(2*R*,5*S*)-5-ethyl-2-methyl-2-propyl-1,3-oxazolidin-3-yl]ethanone

**DOI:** 10.1107/S1600536810016090

**Published:** 2010-05-08

**Authors:** Ying Fu, Fei Ye, Xiaotian Wen

**Affiliations:** a College of Science, Northeast Agricultural University, Harbin 150030, People’s Republic of China

## Abstract

In the title compound, C_11_H_19_Cl_2_NO_2_, the oxazolidine ring is in an envelope conformation with the O atom forming the flap. In the crystal structure, mol­ecules are linked by weak inter­molecular C—H⋯O hydrogen bonds, forming chains.

## Related literature

For general background to *N*-dichloro­acetyl oxazolidine, see: Agami & Couty (2004 ▶); Abu-Qare & Duncan (2002 ▶); Guirado *et al.* (2003 ▶); Davies & Caseley (1999 ▶). For the bioactivity of related compounds, see: Del Buono *et al.* (2007 ▶); Hatzios & Burgos (2004 ▶). For details of the synthesis, see: Fu *et al.* (2009 ▶).
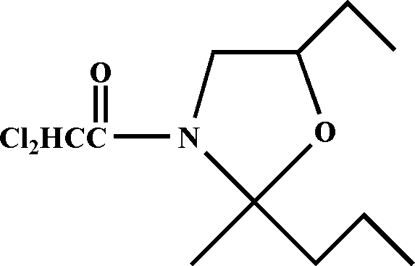


## Experimental

### 

#### Crystal data

C_11_H_19_Cl_2_NO_2_
*M* *_r_* = 268.17
Orthorhombic, 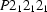

*a* = 6.4834 (12) Å
*b* = 10.795 (2) Å
*c* = 20.030 (4) Å
*V* = 1401.8 (5) Å^3^
*Z* = 4
Mo *K*α radiation
μ = 0.45 mm^−1^
*T* = 293 K
0.32 × 0.24 × 0.20 mm

#### Data collection

Bruker SMART CCD diffractometer
Absorption correction: multi-scan (*SADABS*; Sheldrick, 1996 ▶) *T*_min_ = 0.869, *T*_max_ = 0.915
14134 measured reflections
3499 independent reflections
2117 reflections with *I* > 2σ(*I*)
*R*_int_ = 0.058

#### Refinement

*R*[*F* ^2^ > 2σ(*F* ^2^)] = 0.084
*wR*(*F* ^2^) = 0.258
*S* = 1.02
3499 reflections
148 parameters
H-atom parameters constrained
Δρ_max_ = 0.58 e Å^−3^
Δρ_min_ = −0.39 e Å^−3^
Absolute structure: Flack (1983 ▶) 1468 Friedels
Flack parameter: 0.02 (15)

### 

Data collection: *SMART* (Bruker, 1998 ▶); cell refinement: *SAINT* (Bruker, 1998 ▶); data reduction: *SAINT*; program(s) used to solve structure: *SHELXS97* (Sheldrick, 2008 ▶); program(s) used to refine structure: *SHELXL97* (Sheldrick, 2008 ▶); molecular graphics: *SHELXTL* (Sheldrick, 2008 ▶); software used to prepare material for publication: *SHELXTL*.

## Supplementary Material

Crystal structure: contains datablocks global, I. DOI: 10.1107/S1600536810016090/lh5034sup1.cif

Structure factors: contains datablocks I. DOI: 10.1107/S1600536810016090/lh5034Isup2.hkl

Additional supplementary materials:  crystallographic information; 3D view; checkCIF report

## Figures and Tables

**Table 1:** Hydrogen-bond geometry (Å, °)

*D*—H⋯*A*	*D*—H	H⋯*A*	*D*⋯*A*	*D*—H⋯*A*
C1—H1⋯O2^i^	0.98	2.38	3.327 (5)	163

Symmetry code: (i) 
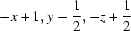
.

**Fig. 1.**
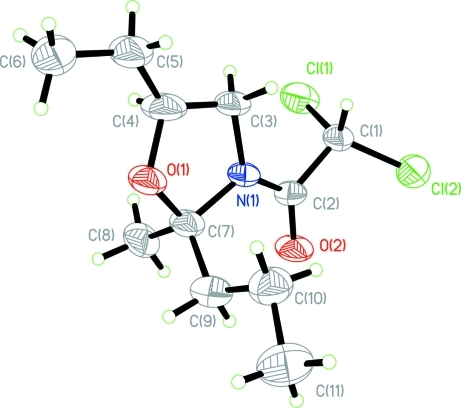
The molecular structure of the title compound, with the atom-labelling scheme. Displacement ellipsoids are drawn at the 30% probability level.

**Fig. 2.**
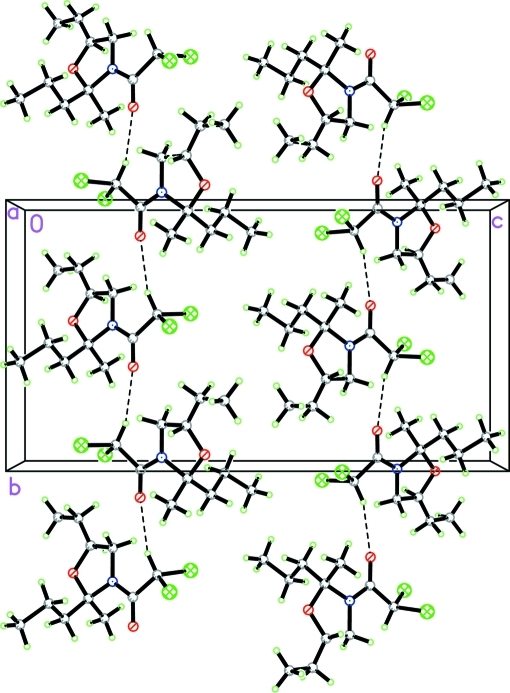
A packing diagram for (I), showing weak hydrogen bonds as dashed lines.

## supplementary crystallographic information

### Comment

N-dichloroacetyl oxazolidines are becoming increasingly important with their excellent biological activity (Agami & Couty, 2004;  Abu-Qare & Duncan, 2002; Guirado *et al.*,  2003; Davies & Caseley, 1999). The discovery of N-dichloroacetyl oxazolidine as a herbicide safener has drawn widespread attention in agricultural biochemistry (Del Buono *et al.*, 2007; Hatzios & Burgos, 2004). As a part of our ongoing investigation of oxazolidine derivatives (Fu *et al.*,  2009) we prepared the title compound.

The molecular structure of the title compound is shown in Fig. 1. In the crystal structure, molecules are linked by weak intermolecular C—H···O hydrogen bonds to form one-dimensional chains (Fig. 2).

### Experimental

The title compound was prepared according to the literature procedure (Fu *et al.*, 2009). The single crystal suitable for X-ray structural analysis was obtained by slow evaporation in petroleum ether and ethyl acetate at room temperature. The title enantiomer spontaneously resolved from a racemic mixture during the crystallization.

### Refinement

All H atoms were initially located in a different Fourier map. The C—H atoms were then constrained to an ideal geometry, with C—H = 0.96-0.98 Å and U_iso_(H) = 1.2U_eq_(C) or 1.5U_eq_(C) for methyl H atoms.

### Crystal data

**Table:**

C_11_H_19_Cl_2_NO_2_	*F*(000) = 568.0
*M**_r_* = 268.17	*D*_x_ = 1.271 Mg m^−^^3^*D*_m_ = 1.271 Mg m^−^^3^*D*_m_ measured by not measured
Orthorhombic, *P*2_1_2_1_2_1_	Mo *K*α radiation, λ = 0.71073 Å
Hall symbol: P 2ac 2ab	Cell parameters from 3142 reflections
*a* = 6.4834 (12) Å	θ = 2.8–20.6°
*b* = 10.795 (2) Å	µ = 0.45 mm^−^^1^
*c* = 20.030 (4) Å	*T* = 293 K
*V* = 1401.8 (5) Å^3^	Block, colourless
*Z* = 4	0.32 × 0.24 × 0.20 mm

### Data collection

**Table:**

Bruker SMART CCD diffractometer	3499 independent reflections
Radiation source: fine-focus sealed tube	2117 reflections with *I* > 2σ(*I*)
graphite	*R*_int_ = 0.058
φ and ω scans	θ_max_ = 28.4°, θ_min_ = 2.8°
Absorption correction: multi-scan (*SADABS*; Sheldrick, 1996)	*h* = −8→8
*T*_min_ = 0.869, *T*_max_ = 0.915	*k* = −14→14
14134 measured reflections	*l* = −25→26

### Refinement

**Table:**

Refinement on *F*^2^	Secondary atom site location: difference Fourier map
Least-squares matrix: full	Hydrogen site location: inferred from neighbouring sites
*R*[*F*^2^ > 2σ(*F*^2^)] = 0.084	H-atom parameters constrained
*wR*(*F*^2^) = 0.258	*w* = 1/[σ^2^(*F*_o_^2^) + (0.165*P*)^2^] where *P* = (*F*_o_^2^ + 2*F*_c_^2^)/3
*S* = 1.02	(Δ/σ)_max_ < 0.001
3499 reflections	Δρ_max_ = 0.58 e Å^−^^3^
148 parameters	Δρ_min_ = −0.39 e Å^−^^3^
0 restraints	Absolute structure: Flack (1983) 1468 Friedels
Primary atom site location: structure-invariant direct methods	Flack parameter: 0.02 (15)

### Special details

**Table:**

Geometry. All esds (except the esd in the dihedral angle between two l.s. planes) are estimated using the full covariance matrix. The cell esds are taken into account individually in the estimation of esds in distances, angles and torsion angles; correlations between esds in cell parameters are only used when they are defined by crystal symmetry. An approximate (isotropic) treatment of cell esds is used for estimating esds involving l.s. planes.
Refinement. Refinement of *F*^2^ against ALL reflections. The weighted *R*-factor wR and goodness of fit *S* are based on *F*^2^, conventional *R*-factors *R* are based on *F*, with *F* set to zero for negative *F*^2^. The threshold expression of *F*^2^ > σ(*F*^2^) is used only for calculating *R*-factors(gt) etc. and is not relevant to the choice of reflections for refinement. *R*-factors based on *F*^2^ are statistically about twice as large as those based on *F*, and *R*- factors based on ALL data will be even larger.

### Fractional atomic coordinates and isotropic or equivalent isotropic displacement parameters (Å^2^)

**Table:**

	*x*	*y*	*z*	*U*_iso_*/*U*_eq_	
C1	0.5074 (7)	0.9192 (4)	0.2071 (2)	0.0675 (11)	
H1	0.4966	0.8365	0.2270	0.081*	
C2	0.5889 (7)	1.0103 (3)	0.2598 (2)	0.0638 (10)	
C3	0.7886 (9)	0.8326 (4)	0.3065 (3)	0.0789 (13)	
H3A	0.6784	0.7758	0.3181	0.095*	
H3B	0.8454	0.8089	0.2636	0.095*	
C4	0.9470 (13)	0.8334 (4)	0.3575 (3)	0.105 (2)	
H4	1.0627	0.8594	0.3293	0.126*	
C5	1.0407 (13)	0.7301 (5)	0.3866 (4)	0.122 (3)	
H5A	1.0922	0.6801	0.3500	0.147*	
H5B	0.9306	0.6828	0.4072	0.147*	
C6	1.2052 (13)	0.7357 (6)	0.4353 (3)	0.111 (2)	
H6A	1.3358	0.7362	0.4127	0.166*	
H6B	1.1976	0.6647	0.4640	0.166*	
H6C	1.1915	0.8098	0.4614	0.166*	
C7	0.8170 (7)	1.0342 (4)	0.3589 (2)	0.0680 (10)	
C8	0.9703 (10)	1.1251 (5)	0.3287 (3)	0.0925 (16)	
H8A	1.0635	1.1533	0.3627	0.139*	
H8B	0.8973	1.1946	0.3104	0.139*	
H8C	1.0470	1.0847	0.2939	0.139*	
C9	0.6750 (10)	1.1009 (5)	0.4051 (3)	0.0875 (14)	
H9A	0.7550	1.1310	0.4427	0.105*	
H9B	0.6205	1.1727	0.3819	0.105*	
C10	0.4982 (11)	1.0277 (6)	0.4315 (3)	0.1013 (18)	
H10A	0.5481	0.9494	0.4488	0.122*	
H10B	0.4023	1.0103	0.3955	0.122*	
C11	0.3855 (15)	1.0989 (8)	0.4874 (4)	0.127 (3)	
H11A	0.4781	1.1119	0.5242	0.190*	
H11B	0.2688	1.0516	0.5023	0.190*	
H11C	0.3396	1.1775	0.4707	0.190*	
Cl1	0.6885 (3)	0.91462 (14)	0.14073 (7)	0.0996 (5)	
Cl2	0.2642 (2)	0.96549 (13)	0.17873 (9)	0.1008 (5)	
N1	0.7143 (5)	0.9597 (3)	0.30507 (18)	0.0629 (8)	
O1	0.9265 (6)	0.9417 (3)	0.39428 (17)	0.0793 (9)	
O2	0.5407 (6)	1.1193 (3)	0.2580 (2)	0.0859 (10)	

### Atomic displacement parameters (Å^2^)

**Table:**

	*U*^11^	*U*^22^	*U*^33^	*U*^12^	*U*^13^	*U*^23^
C1	0.072 (2)	0.0401 (18)	0.090 (3)	0.0031 (17)	−0.013 (2)	0.0002 (18)
C2	0.071 (2)	0.0396 (17)	0.081 (3)	0.0077 (17)	0.000 (2)	−0.0023 (17)
C3	0.093 (3)	0.0354 (18)	0.108 (3)	0.014 (2)	−0.021 (3)	−0.0090 (18)
C4	0.154 (5)	0.046 (2)	0.114 (4)	0.033 (3)	−0.043 (4)	−0.013 (3)
C5	0.149 (6)	0.059 (3)	0.160 (6)	0.031 (4)	−0.071 (5)	−0.020 (3)
C6	0.127 (5)	0.070 (3)	0.134 (5)	0.010 (4)	−0.031 (4)	0.013 (3)
C7	0.084 (3)	0.0379 (17)	0.082 (3)	0.0039 (18)	−0.010 (2)	−0.0029 (16)
C8	0.101 (4)	0.052 (2)	0.124 (4)	−0.020 (3)	−0.013 (3)	0.003 (3)
C9	0.109 (4)	0.060 (3)	0.093 (3)	0.006 (3)	0.003 (3)	−0.010 (2)
C10	0.105 (4)	0.075 (3)	0.124 (5)	0.010 (3)	0.006 (3)	0.000 (3)
C11	0.149 (6)	0.124 (6)	0.107 (4)	0.021 (5)	0.035 (4)	−0.005 (4)
Cl1	0.1169 (11)	0.0780 (8)	0.1038 (9)	−0.0063 (8)	0.0152 (9)	−0.0185 (7)
Cl2	0.0899 (8)	0.0761 (8)	0.1365 (12)	0.0154 (7)	−0.0350 (8)	−0.0093 (7)
N1	0.0715 (19)	0.0298 (13)	0.087 (2)	0.0036 (14)	−0.0060 (17)	−0.0042 (13)
O1	0.102 (2)	0.0413 (14)	0.095 (2)	0.0055 (15)	−0.0232 (19)	−0.0079 (14)
O2	0.114 (3)	0.0338 (13)	0.110 (2)	0.0152 (16)	−0.023 (2)	−0.0013 (14)

### Geometric parameters (Å, °)

**Table:**

C1—C2	1.536 (6)	C6—H6C	0.9600
C1—Cl2	1.750 (4)	C7—O1	1.416 (5)
C1—Cl1	1.775 (5)	C7—C9	1.491 (7)
C1—H1	0.9800	C7—N1	1.501 (5)
C2—O2	1.218 (5)	C7—C8	1.523 (7)
C2—N1	1.335 (6)	C8—H8A	0.9600
C3—C4	1.448 (8)	C8—H8B	0.9600
C3—N1	1.454 (5)	C8—H8C	0.9600
C3—H3A	0.9700	C9—C10	1.490 (9)
C3—H3B	0.9700	C9—H9A	0.9700
C4—O1	1.388 (6)	C9—H9B	0.9700
C4—C5	1.397 (7)	C10—C11	1.542 (9)
C4—H4	0.9800	C10—H10A	0.9700
C5—C6	1.446 (10)	C10—H10B	0.9700
C5—H5A	0.9700	C11—H11A	0.9600
C5—H5B	0.9700	C11—H11B	0.9600
C6—H6A	0.9600	C11—H11C	0.9600
C6—H6B	0.9600		
			
C2—C1—Cl2	110.5 (3)	O1—C7—N1	101.8 (3)
C2—C1—Cl1	107.8 (3)	C9—C7—N1	115.5 (4)
Cl2—C1—Cl1	111.1 (3)	O1—C7—C8	109.0 (4)
C2—C1—H1	109.1	C9—C7—C8	109.8 (4)
Cl2—C1—H1	109.1	N1—C7—C8	110.5 (4)
Cl1—C1—H1	109.1	C7—C8—H8A	109.5
O2—C2—N1	124.9 (4)	C7—C8—H8B	109.5
O2—C2—C1	120.7 (4)	H8A—C8—H8B	109.5
N1—C2—C1	114.5 (3)	C7—C8—H8C	109.5
C4—C3—N1	104.1 (4)	H8A—C8—H8C	109.5
C4—C3—H3A	110.9	H8B—C8—H8C	109.5
N1—C3—H3A	110.9	C10—C9—C7	116.0 (5)
C4—C3—H3B	110.9	C10—C9—H9A	108.3
N1—C3—H3B	110.9	C7—C9—H9A	108.3
H3A—C3—H3B	108.9	C10—C9—H9B	108.3
O1—C4—C5	119.5 (5)	C7—C9—H9B	108.3
O1—C4—C3	108.1 (4)	H9A—C9—H9B	107.4
C5—C4—C3	126.8 (5)	C9—C10—C11	111.0 (6)
O1—C4—H4	97.9	C9—C10—H10A	109.4
C5—C4—H4	97.9	C11—C10—H10A	109.4
C3—C4—H4	97.9	C9—C10—H10B	109.4
C4—C5—C6	124.7 (6)	C11—C10—H10B	109.4
C4—C5—H5A	106.2	H10A—C10—H10B	108.0
C6—C5—H5A	106.2	C10—C11—H11A	109.5
C4—C5—H5B	106.2	C10—C11—H11B	109.5
C6—C5—H5B	106.2	H11A—C11—H11B	109.5
H5A—C5—H5B	106.3	C10—C11—H11C	109.5
C5—C6—H6A	109.5	H11A—C11—H11C	109.5
C5—C6—H6B	109.5	H11B—C11—H11C	109.5
H6A—C6—H6B	109.5	C2—N1—C3	127.0 (3)
C5—C6—H6C	109.5	C2—N1—C7	122.6 (3)
H6A—C6—H6C	109.5	C3—N1—C7	110.1 (3)
H6B—C6—H6C	109.5	C4—O1—C7	112.1 (3)
O1—C7—C9	109.9 (4)		

### Hydrogen-bond geometry (Å, °)

**Table:**

*D*—H···*A*	*D*—H	H···*A*	*D*···*A*	*D*—H···*A*
C1—H1···O2^i^	0.98	2.38	3.327 (5)	163

Symmetry codes: (i) −*x*+1, *y*−1/2, −*z*+1/2.
